# Regulatory T Cells in Autoimmune Hepatitis: Unveiling Their Roles in Mouse Models and Patients

**DOI:** 10.3389/fimmu.2020.575572

**Published:** 2020-10-07

**Authors:** Han Wang, Xinxia Feng, Wei Yan, Dean Tian

**Affiliations:** Department of Gastroenterology, Tongji Hospital, Tongji Medical College, Huazhong University of Science and Technology, Wuhan, China

**Keywords:** autoimmune hepatitis, regulatory T cell, mouse model, cytochrome P450 2D6, treatment

## Abstract

Autoimmune hepatitis (AIH) is a severe and chronic liver disease, and its incidence has increased worldwide in recent years. Research into the pathogenesis of AIH remains limited largely owing to the lack of suitable mouse models. The concanavalin A (ConA) mouse model is a typical and well-established model used to investigate T cell-dependent liver injury. However, ConA-induced hepatitis is acute and usually disappears after 48 h; thus, it does not mimic the pathogenesis of AIH in the human body. Several studies have explored various AIH mouse models, but as yet there is no widely accepted and valid mouse model for AIH. Immunosuppression is the standard clinical therapy for AIH, but patient side effects and recurrence limit its use. Regulatory T cells (Tregs) play critical roles in the maintenance of immune homeostasis and in the prevention of autoimmune diseases, which may provide a potential therapeutic target for AIH therapy. However, the role of Tregs in AIH has not yet been clarified, partly because of difficulties in diagnosing AIH and in collecting patient samples. In this review, we discuss the studies related to Treg in various AIH mouse models and patients with AIH and provide some novel insights for this research area.

## Introduction

Autoimmune hepatitis (AIH) is a progressive inflammatory liver disease characterized by chronic inflammation of the liver, circulating autoantibodies, hypergammaglobulinemia, and specific liver biopsy histologic features (interface hepatitis, rosettes, and lymphocyte invasion) (1, 2). AIH occurs in all ethnicities and can affect children and adults of all ages, with a female predominance (3). AIH is a complex multifactorial polygenic disorder that is thought to be caused by the interaction between triggers and environmental factors in genetically susceptible individuals, leading to the loss of tolerance against one's own liver antigens (4, 5). Standard immunosuppressive treatment approaches have remained static for decades; these treatments are effective in most patients, although their mechanisms are unclear (6). Many patients with AIH must receive long-term immunosuppressive treatment to prevent disease relapse (7).

The precise etiology and pathophysiology of AIH remain largely unknown, and related basic research in this field is relatively limited compared with other types of hepatitis, such as viral hepatitis or steatohepatitis. The complexity of the disease, the difficulty of confirming a clinical diagnosis, and the lack of valid animal models for AIH research contribute to this situation. Researchers have been working on the establishment of a mouse model for AIH for a

long time, but as yet no valid animal model has been widely accepted (8, 9). In 1992, Tiegs et al. (10) established a T cell-mediated acute hepatitis mouse model using concanavalin A (ConA), which is a typical and well-recognized mouse model used to investigate T cell-mediated liver injury. However, hepatitis in mice livers induced by ConA is acute and often disappears after 48 h; furthermore, there is no autoantibody production or liver fibrosis. In 2008, Christen et al. (11) established a chronic AIH mouse model using adenovirus that expressed human cytochrome P450 2D6 (CYP2D6), which is a well-recognized human autoantigen in type-2 AIH. In 2013, Hardtke-Wolenski et al. (12) established an AIH model against FTCD in NOD with one single adenoviral injection into the tail vein, but in 2016 they did not observe chronic AIH with either soluble liver antigen (SLA) or CYP2D6 in B6, Balb/c and FVB (13). Given that a one-time adenovirus injection may not be sufficient to induce a stable and long-term mouse model, we improved the method to establish this CYP2D6-AIH mouse model through the repeated injection of the CYP2D6 plasmid to achieve multiple gene transection in the liver (14). However, although great efforts have been made, the identification of a valid mouse model for use in the AIH research field remains an urgent, unsolved issue.

Regulatory T cells (Tregs) that express the transcription factor Foxp3 play an important role in the maintenance of immunological self-tolerance and the prevention of autoimmunity (15). Tregs are recognized as the body's main source of tolerance regulation, and their impairment has been associated with the development of various autoimmune diseases (16). Given the ability of Tregs to suppress the destructive pro-inflammatory and cytolytic activities of immune effector cells, the adoptive transfer of Treg has been considered as a potential future treatment for AIH (17). However, the role of Tregs in AIH remains controversial (18, 19), and for nearly 20 years, researchers have been exploring whether or not the Tregs are impaired in AIH. The results of this research are inconclusive, partly because of the different mouse models used in the different studies and patient heterogeneity. Here we review these issues by first summarizing the different mouse models and then focusing on the studies of AIH to understand what roles have been played by Tregs in the pathogenesis of AIH.

## AIH: Current Research Status

Autoimmune hepatitis (AIH) is a liver-specific autoimmune disease first described in 1951 (5). AIH manifests in all age groups, and its incidence has shown an increasing trend (20). The disease is now widely known as a consequence of immune tolerance breakdown, leading to an autoimmune response against hepatocytes that induces liver injury (21). AIH is divided into two main types based on the serological profiles of persistent autoantibodies (22). Type 1 AIH is defined by the presence of antinuclear antibodies (ANAs) and/or anti-smooth muscle antibodies (SMAs), while type 2 AIH is defined by the presence of anti-liver-kidney microsomal 1 (LKM-1) antibody and/or anti-liver cytosol type 1 (LC1) antibody (23, 24). The pathogenesis of AIH is complicated and unclear, but it likely involves environmental factors (25), genetic factors, epigenetic factors (26, 27), and a dynamic immunological microenvironment (28). It is widely accepted that both the initiation of the self-attack and the subsequent dysregulation of the immune system in the liver microenvironment contribute to the progressive process of liver damage. During this process, helper T (Th) cells play the most important role in triggering this self-attack process by recognizing the autoantigens (29), while B cells are responsible for the subsequent production of autoantibodies (29). Meanwhile, increasing evidence suggests the emerging role of impaired immunoregulation in this process (30–32). Cells and cytokines involved immunoregulation maintaining the liver immunologic balance to protect the liver from serve damage under inflammatory attack have also been widely studied in recent years (29, 33, 34). However, further studies are required to elucidate their exact roles in the AIH disease process.

Clinically, although a well-established diagnostic scoring system with acceptable specificity and sensitivity is in use (35), AIH diagnosis remains difficult, largely owing to its dependence on liver biopsy. Moreover, due to the insidious and atypical symptoms of patients, AIH is difficult to diagnose in the very early stage, which leads to delays in treatment. Therefore, an increasing number of studies have explored AIH biomarkers that could be applied to obtain an early diagnosis (36, 37). Given the diagnostic difficulties of AIH, the related clinical research is difficult to carry out. Furthermore, there is no effective therapy for patients with AIH except for standard immunosuppressive treatment using corticosteroids with or without azathioprine (2, 38). However, not all patients respond well to this treatment, and most will develop disease relapse after drug withdrawal (39). Immunoregulatory therapy has also been explored as a potential therapy for AIH; however, it has not yet been clinically applied (6, 40, 41). Much research effort has been given in recent decades to comprehensively understand the pathogenesis of AIH and to find potential therapeutic targets (42, 43). However, it is difficult to progress basic research in the area of AIH because of the lack of a suitable and widely accepted mouse model that imitates the AIH disease process in humans.

## Different AIH Mouse Models

### ConA Mouse Model

There has been a lot of research effort in the last 50 years to establish AIH mouse models (44–46). In 1992, Tiegs et al. (10) were the first to report the use of ConA to establish a T cell-mediated hepatitis mouse model, which is now the most widely used tool to investigate immune-mediated liver injury (47–49). Progressive hepatitis, severe lymphocytes infiltration, and significantly increased transaminase release are observed within 8 h; this condition results from the activation of T lymphocytes by macrophages in the presence of ConA. Subsequently, IFN-γ (interferon-γ) and TNF-α (tumor necrosis factor-α) were proven to be the critical mediators of liver injury in ConA-treated mice (50), which is similar to the situation in patients with AIH (51). Over the past decades, substantial basic research on AIH was carried out using this typical and well-established ConA mouse model due to its convenience and low cost (52–54). However, hepatitis in this mouse model is acute and usually disappears within 48 h (10, 55). Characteristic features of AIH, such as the presence of autoantibodies, typical interface hepatitis, and progressive liver fibrosis, are not observed in this model.

### Mouse Model of Type 2 AIH

Autoantibodies against the autoantigens expressed on hepatocytes play crucial roles in the pathogenesis of AIH; therefore, breaking immune tolerance using known autoantigens may provide a pathway for the establishment of a chronic AIH mouse model. In type 2 AIH, CYP2D6 is one of the most thoroughly characterized autoantigens that is recognized by type 1 liver/kidney microsomal autoantibodies (LKM-1) (56), while the target of LC1 antibodies is FTCD (57). In 2004, Lapierre et al. (58) first established a murine model of AIH via DNA immunization against CYP2D6 and FTCD. The authors found cytotoxic-specific T cells and the presence of necroinflammation in the liver, with the alanine aminotransferase (ALT) level peaking at 4 and 7 months post-injection. Anti-LKM1 and anti-LC1 antibodies were also detected in the mice sera, which stay elevated for at least 8 months (59). The genetic background was proven to affect AIH development by the fact that C57Bl/6J mice were more susceptible to the DNA vaccination compared with the 129/Sv or BALB/c strains (60). The age and sex susceptibility bias of AIH was also investigated in this model (61). A few years later, Christen et al. infected mice with adenovirus(Ad) that expressed human CYP2D6 based on an FVB/N or C57Bl/6J background and observed the characteristic pathological features and the presence of anti-LKM1 antibodies in their livers (11, 62–64). In this CYP2D6 model, acute liver damage resulting from the adenovirus infection was first observed, followed by massive infiltration, progressive liver inflammation, and fibrosis at 8 weeks, which is a much shorter timeframe than the model from Lapierre et al. (58).

**Figure 1:**
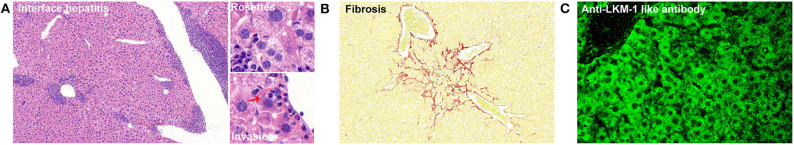
Characteristic features of type 2 AIH in an improved mouse model. **(A)** Chronic liver inflammation (interface hepatitis) and characteristic pathological features (rosettes and lymphocytes invasion) are shown. The red arrow indicates the hepatocyte. **(B)** Sirius red staining showing the fibrosis in a mouse liver. The red color indicates collagenous fibers. **(C)** Stained autoantibodies from the plasma of AIH mice.

In 2013, Hardtke-Wolenski et al. (12) developed a model of type 2 AIH by inducing a self-limited adenoviral infection with FTCD. The authors also demonstrated that the development of AIH in autoantibody-positive animals was determined by the genetic background (13). However, they did not observe the development of AIH when using Christen's protocol and a one-time Ad-CYP2D6 injection on the FVB/N background. These differences may have been due to their different injection methods or the different time points at which the mice were sacrificed. Recently, we also introduced an improved method for establishing a CYP2D6-induced AIH mouse model using an initial one-time adenovirus infection and repeated injections of human CYP2D6 plasmid based on the hydrodynamic-based liver-targeted gene delivery technique (14). This achieves the initial transient hepatitis using pure adenovirus and the multiple continuous expression of the naked CYP2D6 plasmid. Autoantibodies and interface hepatitis can be observed at 4 weeks after the first injection, with the appearance of progressive liver fibrosis at 5 weeks. This provides another technical approach to establishing a CYP2D6-induced type 2 AIH mouse model (Figure 1).

### Other Mouse Models for AIH

**Figure 2:**
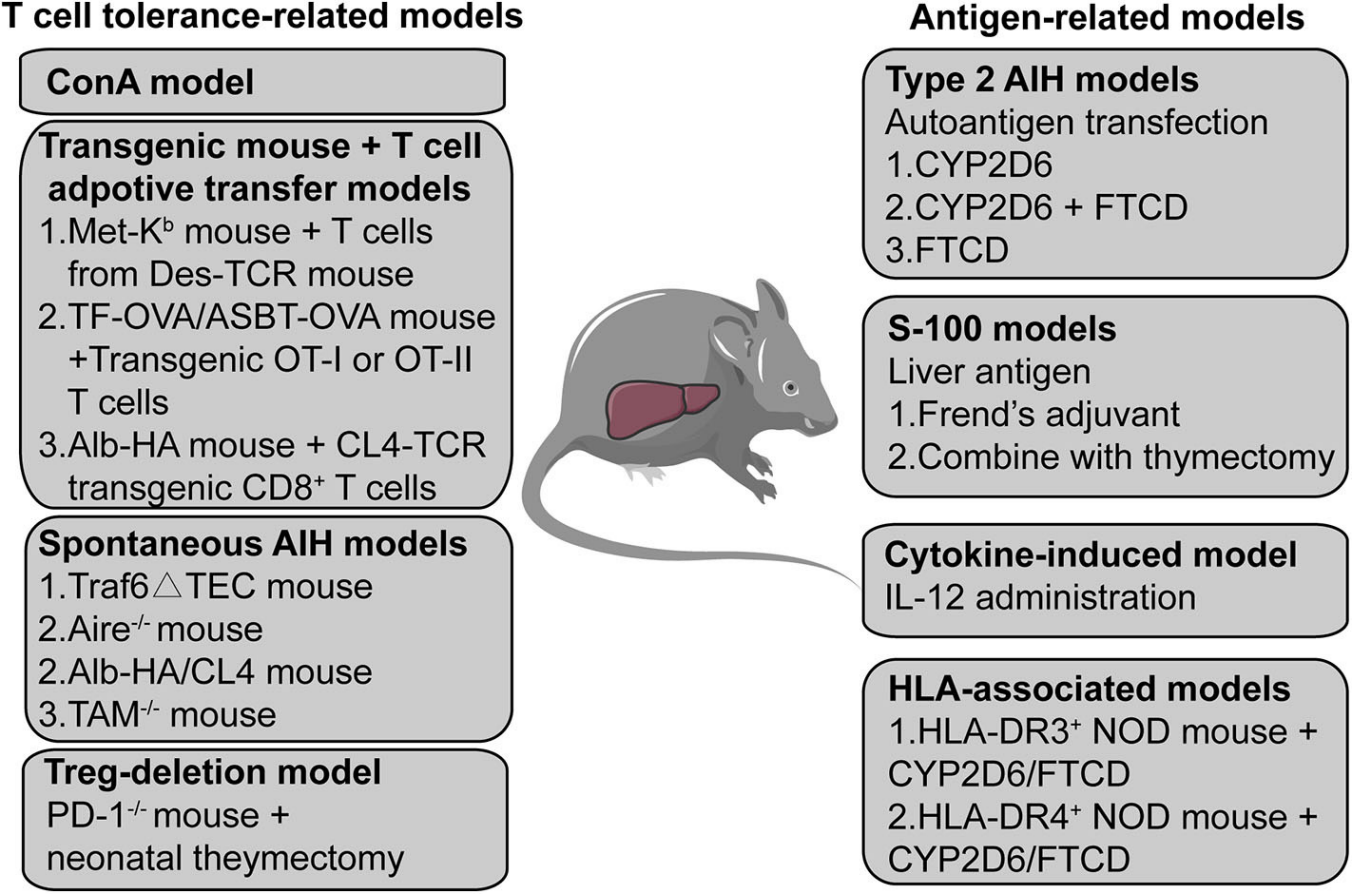
A summary of AIH mouse models. The mouse models are divided into two categories; one is based on a T cell-related mechanism and the other is based on an autoantigen- or liver antigen-related mechanism. The ConA mouse model is the most widely used mouse model to investigate acute T cell-mediated liver injury. Transgenic mice combined with T cell adoptive transfer also provides a method to establish an AIH mouse model. Some transgenic or gene knockout mice can develop spontaneous AIH-like disease. Treg depletion may also function as a potential method to induce AIH in mice. Transfecting the human autoantigen CYP2D6 or FTCD from type 2 AIH into mice may simulate the initiation process of type 2 AIH in humans to establish a chronic type 2 AIH mouse model. S-100, a supernatant of syngeneic liver homogenate, has also been used to induce AIH in mice. The expression of transgenic IL-2 in hepatocytes causes loss of tolerance of hepatocellular antigens, leading to chronic type 1 AIH-like disease in mice. Transfection HLA-DR3 or HLA-DR4 transgenic mice with the non-obese -diabetic background with a plasmid containing CYP2D6 and FTCD can also induce AIH.

In addition to the acute ConA mouse model and the chronic type 2 AIH mouse models, other studies have reported using different methods to mimic AIH in the human body; these are presented in Figure 2. First, liver antigens have been used to initiate AIH by injection of syngeneic liver homogenate (S-100) in complete Freund's adjuvant (46) alone or with thymectomy (65), which can be used to induced AIH in mice. Second, exogenous cytokines such as IL (interleukin)-12 may also be used as triggers to induce AIH (66–68). Third, several transgenic mice that underwent specific T cells adoptive transfer were used to break the peripheral or central tolerance to induce AIH (69–71). The human leukocyte antigen (HLA)-DR3 and DR4 allele related transgenic AIH mouse models have also been established due to their strong genetic association with both type 1 and type 2 AIH (42, 72). Some studies have even reported spontaneous AIH in specific transgenic mouse models (73–75), the mechanism of which largely depends on the breakdown of normal immune tolerance. Furthermore, given that Tregs are the most important regulatory cells maintaining immune tolerance, scientists have also tried to induce AIH in mice by depleting Tregs using PD-1 (programmed cell death protein 1)–/– mice with neonatal thymectomy (76–78).

Although there appear to be many options for AIH mouse models, different models should be carefully selected based on different research aims. To be more specific, the ConA model is more suitable for the investigation of drug-induced liver injury or the therapeutic effect of certain reagents in controlling the acute immune-mediated liver injury. The models based on transgenic/knockout mice (plus T cell transfer/deletion or not) may not suitable for studying particular immune cells due to their deficient immune system even before AIH initiation. Therefore, spontaneous type 2 AIH in mice that triggered by natural disease antigens can better simulate the natural process of AIH in the human body. It will be more consistent with its clinical characteristic as well, which is more appropriate for investigating the roles of immune cells in the disease process of AIH.

## The Role of Tregs in AIH

### Tregs in AIH Mouse Models

Tregs are important regulatory cells that maintain immune tolerance and have shown considerable potential in treating multiple autoimmune diseases (79–81). The depletion of Tregs in mice has been reported as a method to establish an AIH mouse model (76). However, the exact role of Tregs in AIH remains unclear. Scientists have explored the function and number of Tregs in different AIH mouse models, which are summarized in Table 1. The conclusions of these studies are conflicting, probably because of the different mechanisms used to induce AIH in the different mouse models and the different markers and methods used to detect Tregs.

**Table 1:** The quality and function change of Treg in AIH mouse model.

**Mouse model**	**Strain (gender)**	**Marker of Treg (detection methods)**	**Peripheral**		**Liver**		**Year (Reference)**
			**Number**	**Function**	**Number**	**Function**	
ConA mouse model	C57BL/6J (male)	CD4^+^Foxp3^+^(FC);Foxp3(WB;PCR)	(–)	(–)	↑	(–)	2018 (81)
S100 liver homogenate mouse model	C57BL/6J (male)	CD4^+^CD25^+^Foxp3^+^(FC);Foxp3(PCR)	↑	(–)	↑	(–)	2018 (82)
S100 liver homogenate mouse model	C57BL/6J (female)	CD4^+^Foxp3^+^(FC);Foxp3(PCR)	↓	(–)	(–)	(–)	2018 (83)
TF-OVA mouse + Transfer of TCR tg OVA-specific T cells	C57Bl/6J (–)	CD4^+^CD25^+^Foxp3^+^(FC);Foxp3(IHC)	→	(–)	↑	→	2015 (84)
Aire^−/−^ mouse model	BALB/c (female)	CD4^+^ CD25^+^Foxp3^+^(FC)	→	→	↑	↓	2015 (74)
ConA mouse model	C57BL/6J (male)	CD4^+^CD25^+^(FC)	(–)	(–)	↑	(–)	2014 (85)
Traf6ΔTEC mouse model	C57Bl/6J (–)	CD4^+^Foxp3^+^(FC)	(–)	(–)	↑	(–)	2013 (73)
Type 2 AIH mouse model	C57BL/6J (female)	CD4^+^CD25^+^Foxp3^+^(FC)	↓	(–)	↓	↓	2013 (86)
ConA mouse model	C57BL/6J (female)	CD4^+^Foxp3^+^(FC);Foxp3(WB;PCR)	→	(–)	↑	(–)	2008 (87)
PD-1^−/−^+ NTx mouse model	BALB/c (–)	CD4^+^Foxp3^+^(FC)	↓	(–)	(–)	(–)	2008 (75)
ConA mouse model	C57BL/6J (male)	CD4^+^Foxp3^+^(FC)	↑	↑	↑	↑	2007 (88)

*AIH, autoimmune hepatitis; ConA, concanavalin A; S100, syngeneic liver homogenate; TF-OVA mouse, the model antigen ovalbumin is expressed in hepatocytes of the mouse; Traf6ΔTEC, conditional deletion of tumor necrosis factor receptor-associated factor 6 expression in the thymic epithelial cells; PD-1, programmed cell death protein 1; NTx, neonatal theymectomy; FC, flow cytometry; WB, western blotting; PCR, polymerase chain reaction; IHC, immunohistochemistry*.

For instance, Tregs behave completely differently in acute and chronic mouse models. In the ConA mouse model, the function and frequency of Tregs are increased both in the liver and in the peripheral environment (85–88), but decreased in the type 2 AIH mouse model (82). The increased Tregs in the liver of the ConA mouse model are mainly responding to the severe liver inflammation induced by ConA, while their deficiency results in long-term impaired immune homeostasis in the chronic type 2 mouse model. Moreover, two different studies reported contrary results regarding Tregs in the liver of the S100 liver homogenate mouse model (83, 89). This discrepancy likely arose because of the different sexes of the experimental mice, as discussed in a previous study (61). However, using a drug-induced AIH mouse model, one study showed that IL-33-induced Tregs confer protection against liver damage in both female and male mice (90). Furthermore, several studies have indicated that Tregs behave differently inside and outside of the hepatic microenvironment. Alexandropoulos et al. reported evidence of Tregs-mechanisms in the liver based on the Traf6ΔTEC mouse model, but the peripheral tolerance in the mice was normal (74). Schott et al. found that Tregs accumulated in the liver of the TF-OVA mouse model but remained unchanged in the spleen (84). The different prevalence of Tregs in the mouse liver and peripheral microenvironment (spleen or blood) may reflect the unique and organ-specific pathogenesis of AIH.

Although Tregs show different changes among the different models, some studies have focused on efforts to improve Tregs immunoregulation in mice. In 2015, Hardtke-Wolenski et al. (75) reported hyperproliferative intrahepatic Tregs in a spontaneous transgenic AIH mouse model with ongoing severe AIH, and they also found the AIH in those mice could be treated by adoptive Tregs transfer. Those findings suggest that the intrahepatic Tregs that increase during the process of AIH are dysfunctional or not sufficient to control the severe liver inflammation. Some other studies have also provided evidence for the curative effect of Tregs adoptive transfer in different AIH mouse models (82, 86, 91). Several animal studies have investigated improving the Tregs frequency (88) or the impaired Treg/Th17 balance (83, 85, 92) in the liver to reduce immune-mediated liver damage in mice. In general, owing to the different mechanisms in the different mouse models to trigger AIH, it is reasonable to have incoherent observation results from various studies. However, the various Treg-related studies in different mouse models can still provide us with this consistent enlightenment—improving the Tregs immunoregulation (function and/or number) in AIH can relieve the hepatitis and liver damage in mice.

### Tregs in Patients With AIH

Over the past 20 years, Tregs in patients with AIH have been investigated, but the function and number of Tregs in these patients remains controversial (Table 2). The conflicting results may be due to patient heterogeneity, the use of different markers, and differences in the methodologies applied in various studies. It was long believed that in AIH, Tregs were functionally and/or numerically impaired (18, 32, 95, 97–99). Several studies suggested that decreases of suppressive molecules or genes in patients with AIH may lead to Tregs deficiency (96, 99, 100). In contrast, recent studies have shown that Tregs in patients with AIH are fully functional and are not reduced in frequency (19, 31). Moreover, some studies have even shown aggregated Tregs in patients with AIH (19, 89, 94). A study reported the isolated Tregs from the peripheral blood of patients with AIH are suppressive, possess the functional markers CD39 and CTLA-4, and express the C-X-C chemokine receptor (CXCR3) (101). As mentioned above for the mouse model, altered levels of Tregs inside and outside of the liver have also been found in patients with AIH (19).

**Table 2:** The quality and function change of Treg in patients with AIH.

**Patients group (number)**	**Control group (number)**	**Patients type**	**Marker of Treg (detection methods)**	**Peripheral blood**		**Liver**		**Year (Reference)**
				**Number**	**Function**	**Number**	**Function**	
AIH (*n* = 32)	HC (*n* = 20)	Adults	CD4^+^CD25^+^Foxp3^+^ (FC)	↑	(–)	(–)	(–)	2018 (83)
AIH (*n* = 42)	HC (*n* = 15)	(−)	CD4^+^CD25^hi^CD127^lo/−^Foxp3^+^ (FC)	→	(–)	(–)	(–)	2017 (31)
pAIH (*n* = 40)	aAIH (*n* = 45)	Children and adults	CD4^+^Foxp3^+^(IF)	(–)	(–)	↑	(–)	2017 (93)
AIH (*n* = 50)	Other chronic liver disease (*n* = 50)	Children	CD4^+^Foxp3^+^ (Tissue immunostaining)	(–)	(–)	↑	(–)	2016 (94)
AIH and ASC (*n* = 43)	HC (*n* = 22)	Adults and children	CD4^+^CD25^hi/+^CD127-(FC)	↓	↓	(–)	(–)	2015 (32)
AIH (*n* = 77)	HC (*n* = 42) or NASH (*n* = 8)	Adults	CD4^+^CD25^hi^CD127^lo^Foxp3^+^(FC); Foxp3(IHC)	→ (vs. HC)	→ (vs. HC)	↑ (vs. NASH)	(–)	2012 (19)
Type 1 AIH (*n* = 47)	HC (*n* = 28)	Adults	CD4^+^CD25^hi^(FC); Foxp3^+^(IHC)	↓	↓	↓	(–)	2010 (95)
Type 1 AIH (*n* = 15)	HC (*n* = 9)	Adults	CD4^+^CD25^+^(FC)	↑	↓	(–)	(–)	2008 (96)
AIH (*n* = 25)	HC (*n* = 15)	Adults and children	Foxp3(PCR)	↓	(–)	(–)	(–)	2006 (18)
AIH (*n* = 28)	HC (*n* = 15)	Adults and children	CD4^+^CD25^+^(FC)	(–)	↓	(–)	(–)	2005 (97)
AIH (*n* = 41)	HC (*n* = 18)	Adults and children	CD4^+^CD25^+^(FC)	↓	→	(–)	(–)	2004 (98)

*AIH, autoimmune hepatitis; pAIH, pediatric AIH; aAIH, adult AIH; ASC, autoimmune hepatitis-sclerosing cholangitis; HC, healthy control; NASH, non-alcoholic hepatitis; FC, flow cytometry; PCR, polymerase chain reaction*.

Meanwhile, Tregs seem to behave differently between adult and pediatric patients with AIH. Unlike adult patients, a numerical Tregs defect has long been considered in pediatric patients. However, recent studies showed enriched intrahepatic Tregs in pediatric AIH (93, 94). Moreover, the increase of Treg/total T cells was reported to be even more pronounced in pediatric AIH than in adult AIH due to fewer infiltrating T and B cells (93). Overall, given the severe inflammatory microenvironment in the liver of patients with AIH, the enrichment of Tregs is probably because of their recruitment from the periphery via various chemokine pathways. However, whether the Tregs can maintain their full function in the special hepatic microenvironment in AIH remains unknown. Although the Tregs isolated from the peripheral blood of patients with AIH can suppress the proliferation of effector T cells *in vitro*, the intrahepatic Tregs are the ones that modulate the immune microenvironment in the liver. Although isolating Tregs from the liver of patients is challenging, we can still speculate that the Tregs in the liver in AIH are impaired or not sufficient to control the inflammation.

As mentioned, the current management of AIH involves administering corticosteroids alone or in combination with azathioprine (102). Most patients achieve remission (103), but up to 90% develop a disease relapse after therapy withdrawal (38, 104, 105). Notably, the Tregs frequency has been reported as being significantly higher in patients with active AIH than in those who are in a state of remission (19). Moreover, patients with AIH who are untreated appeared to have a higher frequency of Tregs in the blood compared to patients under treatment (19, 93), which may be caused by a decrease of IL-2 levels (32). The disproportional decrease of intrahepatic Tregs during therapy might explain the high relapse rates after discontinuation of immunosuppressants (106), which suggests that increasing intrahepatic immunoregulation may be a better treatment strategy for the long-term control of AIH (97). Low-dose IL-2 has been suggested as a treatment for type 2 AIH and it can result in the expansion of Tregs (41, 107). Blocking Th17 is also a potential avenue for therapy development because it can converts Tregs into a more suppressive phenotype (108). Moreover, growing evidence supports Tregs adoptive transfer as a novel and effective mode of treatment for autoimmune diseases (80, 109, 110). Functionally enhanced Tregs can be expanded and generated *de novo* in patients with AIH (111). The generation of antigen-specific Tregs may represent a superior therapy option due to its more specific and potent suppressive function for effector T cells (112, 113). Based on the above discussion, enhanced Tregs adoptive transfer may open an avenue for long-term AIH treatment.

### Interaction of Treg With Other Cells in AIH

**Figure 3:**
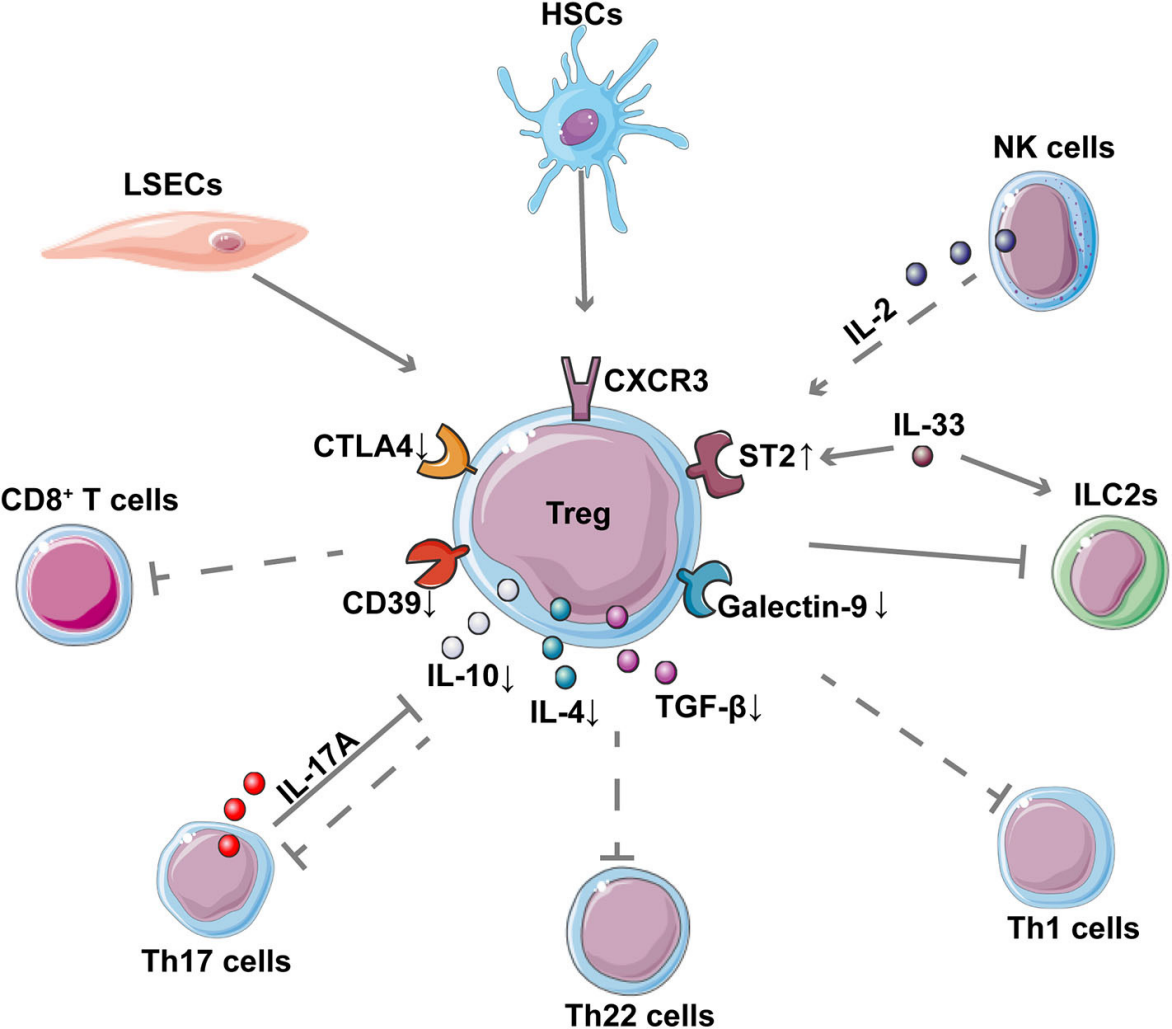
Interaction between Tregs and other cells in AIH. The network shows the interaction of Tregs with other cells as well as the down-regulation of important inhibitory molecules and cytokines in Tregs of patients with AIH. The dotted lines represent the reported decreased regulation in AIH. CD8+ T cells, Th17 cells, Th22 cells, and Th1 cells contribute to inflammatory liver injury in AIH; these cells, are suppressed by Tregs. LSECs and NK cells contribute to the expansion of Tregs while HSCs can enhance the suppressive function of Tregs in AIH. Th17 is reported to inhibit Treg through IL-17A. IL-33 can enhance the expression of ST2 on the surface of Treg, thereby regulating the pro-inflammatory ILC2s in immune-mediated hepatitis.

AIH can be described as a clinical syndrome of the immune-mediated destruction of hepatocytes. The processes of various immune and non-immune cells in the hepatic microenvironment affect each other, forming an interacting network. Tregs, the main regulators of the immune system, can work with other cells in the liver, contributing to the disease process of immune-mediated liver injury (Figure 3). It is well-documented that an imbalance between Tregs and effector T cells is related to AIH pathogenesis (114, 115). The Treg/Th17 ratio has been regarded as a predictor of the degree of liver inflammation, as well as a therapeutic target in AIH (30, 83, 85, 89, 116). The inability of Tregs to efficiently suppress IL-17 production by Th17 cells may be crucial to the pathogenesis of AIH (100). In turn, inhibiting IL-17 has been proven to increase the expression of Foxp3 by CD25^−^ cells (ngTregs), which allows ngTregs to differentiate into functionally stable immune inhibitory cells (108). Additionally, Tregs in patients with AIH are reportedly unable to regulate CD8^+^ T cell proliferation and cytokine production, which may contribute to the initiation of AIH damage.

The crosstalk of innate immune cells and Tregs has also been reported recently. A clinical study of type 2 AIH showed that natural killer (NK) cells display an altered cytokine pattern characterized by increased IFN-γ and reduced IL-2 production, which can contribute to impaired Tregs function (107). Using a Con A mouse model, a study indicated that the IL-33-elicited hepatic ST2^+^ Tregs might counteract the inflammatory activity of type 2 innate lymphoid cells (ILC2s), which participate in the pathogenesis of immune-mediated hepatitis (117).

Some non-immune cells in the liver may also affect the behavior of Tregs in AIH. For example, liver sinusoidal endothelial cells (LSECs) were reported to prime CD4^+^ T cells into a CD45RB^low^ memory phenotype, which might belong to the expanding group of Foxp3^−^ Tregs in the TF-OVA mouse model (118). Moreover, Huang et al. (91) also found that hepatic stellate cells (HSCs) can stimulate allogeneic Tregs proliferation and can also enhance the suppressive activity of Tregs to inhibit the proliferation of effector T cells in the ConA mouse model. An altered cytokines profile secreted by Tregs in AIH, such as IL-10, IL-4, and transforming growth factor β (TGF-β), and their decreased expression of inhibitory molecules such as cytotoxic T-lymphocytes-associated protein 4 (CTLA-4) and CD39 has also been reported (96, 100). These changes can influence the direct and indirect interactions of Tregs with other target cells. In addition, one study indicated that the reduced levels of T cell immunoglobulin and mucin domain 3 (Tim-3) on CD4^+^CD25^−^ effector cells and of galectin-9 in Tregs contributes to the impaired immunoregulation in patients with AIH by enabling effector T cells to evade Tregs (99).

## Tregs in Other Autoimmune Liver Diseases

Studies in other autoimmune liver diseases, such as primary biliary cirrhosis (PBC) or primary sclerosing cholangitis (PSC), have also provided evidence that the intrahepatic Tregs may be affected by the unique microenvironment of AIH. Unlike in AIH, peripheral and intrahepatic Tregs have long been well-documented to be numerically and functionally defective in patients with PBC and murine models of PBC (119–121). Gershwin et al. has confirmed the important role of Treg deficiency in the initiation of PBC, which may be due to the loss of the IL-2 receptor alpha (IL2RA) gene (122–124). They also achieve successful immunotherapy of PBC by adoptive transfer of Tregs (125). Moreover, a patient study revealed the level of Tregs was markedly lower in affected PBC portal tracts compared with AIH, while the CD8^+^T cell/FoxP3^+^ Treg ratio was significantly higher in the livers of late-stage PBC compared with early-stage AIH (126). Compared to PBC, the number of intrahepatic Tregs was reported to be even lower in PSC, which is also associated with the IL2RA gene (127). One study indicated that Tregs adoptive transfer and neutralization of IL-12 may be a treatment strategy to control the cholangitis in PSC (128). Therefore, a reduced number of Tregs has long been regarded as an initiating and promoting factor of the disease processes of PBC and PSC. Compared to PBC and PSC, the increased intrahepatic Tregs in AIH seem to be a consequence rather than a driver of the disease process. This hypothesis can explain why the increased Tregs cannot control the inflammation while the adoptive transfer of *in vitro* enhanced Tregs can relieve AIH.

## Current Treg-Based Therapy in Autoimmune Diseases

Growing evidence shows the excellent safety and therapeutic effect of Treg-based therapy. There have been 51 clinical trials related to Treg-based therapy, with 12 of these trials related to autoimmune diseases (129). Clinical trials performed in patients with type 1 diabetes (T1D) (109, 110) demonstrated the efficacy of Treg therapy in these patients, with no infusion reactions or cell therapy-related high-grade adverse events. A case report described a patient with systemic lupus erythematosus (SLE) treated with autologous adoptive Treg cell therapy (80). The patient initially developed a transient but then a sustained increase in the percentage of highly activated Tregs. A recent study also reported that three patients with amyotrophic lateral sclerosis (ALS) benefited from expanded autologous Treg infusions (130). Several clinical trials have been initiated to assess the safety and efficacy of Treg therapy in other autoimmune diseases such as inflammatory bowel disease, Guillain–Barré syndrome, pemphigus vulgaris, and Alzheimer's disease (129). Given the important role of IL-2 in Treg survival and expansion and the better availability of IL-2 *in vitro*, clinical trials of low-dose IL-2 in various autoimmune diseases have been undertaken (41, 131–134). A phase I clinical trial investigating Treg-based therapy has also been initiated in AIH (NCT02704338) (129). However, much more research effort is needed before adoptive Treg therapy can be clinically translated to patients with AIH.

## Conclusions and Future Perspectives

AIH is a chronic and progressive immune-mediated liver disease that can lead to cirrhosis, hepatocellular carcinoma, liver transplantation, and death (135–137). The pathogenesis of AIH largely remains unclear, while the main clinical treatment consists of immunosuppressive therapy. Although most patients respond well to immunosuppressants, the disease relapses after withdrawal. Animal models are crucial tools to better understand the pathogenetic mechanisms of AIH and to identify potential therapeutic targets. As yet, there is no widely accepted AIH mouse model for this research field. The chronic type 2 AIH mouse model might be a better option to simulate the natural disease process of AIH. Tregs therapy is now emerging as a potential treatment route for a wide variety of autoimmune diseases, while the efficacy of this therapy in AIH remains unclear. Although there is an increased understanding of the roles of Tregs in animal models and patients with AIH, whether their intrahepatic Tregs are impaired or not is not clear. The fact that the increased number of Tregs in the liver of patients with AIH are unable to control the hepatitis indicates the impaired function of Tregs in the special hepatic microenvironment of AIH. However, even though AIH is not driven by the impairment of Tregs, Treg-based therapies for AIH might be effective, providing a potential avenue for the long-term control of AIH.

## Author Contributions

DT designed the topic. HW wrote the manuscript. XF generated figures and tables. WY helped to review and edited the manuscript. All authors contributed to the article and approved the submitted version.

## Conflict of Interest

The authors declare that the research was conducted in the absence of any commercial or financial relationships that could be construed as a potential conflict of interest.

## Abbreviations

AIH: autoimmune hepatitis
ConA: concanavalin A
CYP2D6: cytochrome P450 2D6
FTCD: formiminotransferase cyclodeaminase
Treg: regulatory T cell
ANA: antinuclear antibodies
SMA: anti-smooth muscle antibodies
LKM-1: anti-liver-kidney microsomal 1
LC1: anti-liver cytosol type 1
Th cells: T-helper cells
ALT: alanine aminotransferase
Ad: adenovirus
S-100: syngeneic liver homogenate
HLA: human leukocyte antigen
PD-1: programmed cell death protein 1
Ntx: neonatal theymectomy
Met-Kb mouse: mouse express class I gene (H-2Kb) under the control of metallothionein promoter
Des-TCR mouse: mouse expresses on 98% of their CD8+ T cells a TCR recognizing a complex formed by the class I gene (H-2Kb) molecule and self-peptides
TF-OVA mouse: the model antigen ovalbumin is expressed in hepatocytes of the mouse
ASBT-OVA mice: the model antigen ovalbumin is expressed in cholangiocytes of the mouse
OT-I T cell: CD8+ T cell
OT-II T cell: CD4+ T cell
Alb-HA mouse: mouse expresses the influenza virus hemagglutinin (HA) autoantigen under control of mouse albumin regulatory elements and alpha-fetoprotein enhancers (Alb) specifically in the liver
CL4-TCR mouse: mouse expresses the clone 4/T-cell receptor
Traf6ΔTEC: conditional deletion of tumor necrosis factor receptor-associated factor 6 expression in the thymic epithelial cells
TAM–/– mouse: mouse that is deficient in Tyro3, Axl, and Mer receptor tyrosine kinases
IL: interleukin
IFN-γ: interferon-γ
TFN-α: tumor necrosis factor-α
TGF-β: transforming growth factor β
CXCR3: C-X-C chemokine receptor
NK cell: natural killer cell
ILC2: type 2 innate lymphoid cell
LSEC: liver sinusoidal endothelial cell
HSC: hepatic stellate cell
Teff: effector T cell
ASC: autoimmune hepatitis-sclerosing cholangitis
HC: healthy control
CTLA-4: T-lymphocytes-associated protein 4
Tim-3: T cell immunoglobulin and mucin domain 3
PBC: primary biliary cirrhosis
PSC: primary sclerosing cholangitis
T1D: type 1 diabetes
SLE: systemic lupus erythematosus
ALS: amyotrophic lateral sclerosis
FC: flow cytometry
WB: western blotting
PCR: polymerase chain reaction
IHC: immunohistochemistry.
